# Association of autoimmune diseases with the occurrence and 28-day mortality of sepsis: an observational and Mendelian randomization study

**DOI:** 10.1186/s13054-023-04763-5

**Published:** 2023-12-05

**Authors:** Hui Li, Xiaojun Pan, Sheng Zhang, Xuan Shen, Wan Li, Weifeng Shang, Zhenliang Wen, Sisi Huang, Limin Chen, Xu Zhang, Dechang Chen, Jiao Liu

**Affiliations:** 1 Department of Critical Care Medicine, Ruijin Hospital, Shanghai Jiao Tong University School of Medicine, Shanghai, 201801 China; 2 Department of General Medicine, Qujiang Town Health Hospital, Fengcheng, Jiangxi China; 3 Center for Reproductive Medicine, Women and Children’s Hospital of Chongqing Medical University, Chongqing, China; 4 Center for Reproductive Medicine, Chongqing Health Center for Women and Children, Chongqing, China; 5 Chongqing Reproductive Genetics Institute, Chongqing, China

**Keywords:** Autoimmune diseases, Sepsis, 28-day mortality, Mendelian randomization, MIMIC-IV

## Abstract

**Background:**

Observational studies have indicated a potential association between autoimmune diseases and the occurrence of sepsis, with an increased risk of mortality among affected patients. However, whether a causal relationship exists between the two remains unknown.

**Methods:**

In the Mendelian randomization (MR) study, we accessed exposure Genome-wide association study (GWAS) data from both the MRC Integrative Epidemiology Unit (MRC-IEU) and the FinnGen consortium. GWAS data for sepsis and its 28-day mortality were obtained from MRC-IEU. We employed univariable, multivariable, and reverse MR analyses to explore potential associations between autoimmune disorders and sepsis and its 28-day mortality. Additionally, a two-step mediation MR analysis was performed to investigate indirect factors possibly influencing the relationship between autoimmune disorders and sepsis. Afterward, we conducted an observational analysis to further explore the relationship between autoimmune disease and occurrence as well as 28-day mortality of sepsis using a real-world database (the MIMIC-IV database). A cohort of 2537 patients diagnosed with autoimmune disease were extracted from the database for analysis. Multivariable logistic regression models were used to confirm the association between autoimmune diseases and the occurrence of sepsis, as well as the 28-day mortality associated with sepsis.

**Results:**

In univariable MR analysis, there appeared to be causal relationships between genetically predicted type 1 diabetes (OR = 1.036, 95% CI = 1.023–1.048, *p* = 9.130E-09), rheumatoid arthritis (OR = 1.077, 95% CI = 1.058–1.097, *p* = 1.00E-15) and sepsis, while a potential causal link was observed between celiac disease and sepsis (OR = 1.013, 95% CI = 1.002–1.024, *p* = 0.026). In a subsequent multivariable MR analysis, only rheumatoid arthritis was found to be independently associated with the risk of sepsis (OR = 1.138, 95% CI = 1.044–1.240, *p* = 3.36E-03). Furthermore, there was no causal link between autoimmune disorders and 28-day mortality from sepsis. In reverse MR analysis, sepsis was suggested to potentially trigger the onset of psoriasis (OR = 1.084, 95% CI = 1.040–1.131, *p* = 1.488E-04). In the real-world observational study, adjusting for multiple confounders, rheumatoid arthritis (OR = 1.34, 95% CI = 1.11–1.64, *p* = 0.003) and multiple sclerosis (OR = 1.31, 95% CI = 1.03–1.68, *p* = 0.02) were associated with a higher risk of sepsis. In addition, we did not find that autoimmune diseases were associated with 28-day mortality from sepsis.

**Conclusion:**

Both in observational and MR analysis, only rheumatoid arthritis is highly correlated with occurrence of sepsis. However, autoimmune disease was not associated with an increased 28-day mortality in patient with sepsis. Sepsis may increase the risk of developing psoriasis.

**Supplementary Information:**

The online version contains supplementary material available at 10.1186/s13054-023-04763-5.

## Introduction

Autoimmune diseases are a group of diseases in which the immune system mounts an immune response against its own normal tissue components, often resulting in chronic tissue and organ damage, affecting approximately 7.6–9.4% of the global population [1]. The primary features of autoimmune diseases include the production of self-targeting antibodies and abnormalities in the function of immune cells. Often, the management of these conditions involves the use of immunomodulatory or immunosuppressive medications, which can result in compromised immune function and an elevated risk of infections [2]. Although retrospective analyses of autoimmune diseases have primarily associated patients with respiratory infections, it is important to highlight that the main drivers of ICU admissions and mortality in this group are severe infections [3, 4]. The evolving environmental changes brought about by societal industrialization have contributed to an increasing incidence of autoimmune diseases. Consequently, the associated risk of pathogenic infections is expected to rise as well [5, 6]. Therefore, prioritizing infection risks in individuals with autoimmune diseases is crucial for mitigating the emergence of life-threatening infectious conditions.

Sepsis is a complex, infection-induced systemic inflammatory response disorder characterized by an imbalance, often accompanied by acute organ dysfunction and a high mortality rate [7]. Despite a decline of 37.0% in the age-standardized incidence of sepsis and a 52.8% decrease in mortality, the burden of this severe condition persists, particularly in developing countries [8, 9]. It is noteworthy that the increased overall burden could be attributed to severe infections resulting from autoimmune diseases and their associated treatments, such as the use of corticosteroids [10]. Some patients with autoimmune diseases were admitted to the intensive care unit at the initial diagnosis [11–14]. Among these cases, sepsis (severe infection) stands out as the primary cause of ICU mortality, followed by acute disease exacerbations [15]. The relationship between autoimmune diseases and sepsis has long been a subject of interest [2]. Therefore, we extracted information on patients with autoimmune diseases from the MIMIC-IV database to explore whether autoimmune diseases increase occurrence of sepsis and the 28-day mortality of sepsis. However, due to limitations in retrospective research, such as potential confounders and selection bias, a consistent conclusion regarding the relationship between autoimmune diseases and sepsis has not been reached [16, 17]. To further investigate the relationship between autoimmune diseases and the incidence and the 28-day mortality of sepsis, we designed a Mendelian randomization (MR) study to overcome the limitations of retrospective research.

MR is an epidemiological technique designed to enhance causal inference [18]. This approach offers two key advantages: It minimizes confounding factors and reduces the likelihood of reverse causation. Genetic variations are randomly allocated during conception and remain unaffected by the development and progression of the disease [19]. Through the implementation of a MR study, we aim to assess the relationship between autoimmune diseases and sepsis, along with the 28-day mortality. This endeavor seeks to further ascertain whether there exists a causal link between genetic variations inherent to distinct autoimmune diseases and the occurrence of sepsis, as well as an elevated risk of mortality within 28-days. In addition, we designed a two-step mediation analysis to examine whether autoimmune diseases can promote sepsis by influencing mediating factors. Furthermore, to enhance the reliability of result inferences, we conducted multivariable MR analyses to correct for associations among different diseases. Following the onset of sepsis, there is often a reshaping of the immune system's functionality. However, whether this reshaping involves long-term, chronic changes in immune function remains unclear. Thus, we have also employed reverse MR to evaluate whether the occurrence of sepsis increases the risk of subsequent autoimmune diseases.

## Methods

### Mendelian randomization

#### Study design and genetic instrument selection

**Fig. 1:**
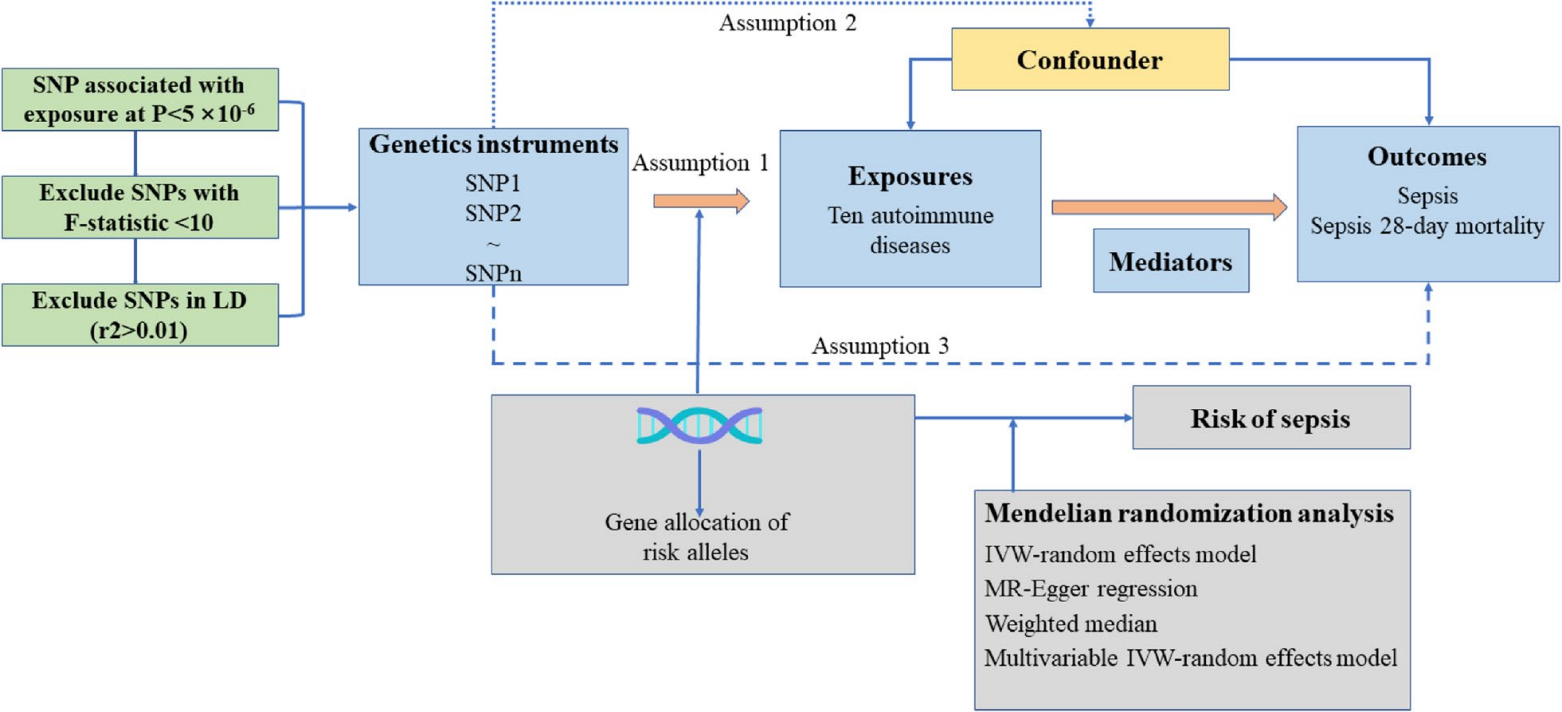
Overview and assumptions of the Mendelian randomization study design

Figure 1 shows the study design and the assumptions of MR in our study [20]. We used publicly available summary statistics from Genome-wide association study (GWAS) sources of predominantly European origin. All studies had current ethical clearance from their respective institutional review boards, including written informed consent from participants and strict quality control. As all analyses herein are based on publicly available summary data, no ethical approval from institutional review boards was required for this study. Three basic assumptions are required for the genetic variants to qualify as valid instrumental variables (IVs): (1) they should be robustly associated with the exposure; (2) they should not be associated with potential confounders of the exposure-outcome association; and (3) they should not influence the outcome by any variable other than the exposure [20]. To validate the initial MR hypothesis, we utilized independent single nucleotide polymorphisms (SNPs) that exhibited a robust association with the exposure, reaching genome-wide significance (*P* < 5 × 10^–6^). These SNPs were carefully chosen to ensure minimal linkage disequilibrium (*r*^2^ < 0.01) within a clump window larger than 5000 kb, thus ensuring their independence. If we follow the same inclusion criteria, the exposure of reverse MR analysis includes too few SNPs, so we have adopted the following criteria. The reverse MR analyses inclusion criteria for the instrumental variable SNP were as follows: *P* < 1 × 10^–5^, *r*^2^ < 0.001 within a clump window larger than 10,000 kb. To further refine the first hypothesis, we quantified the proportion of phenotypic variation explained by the entire set of SNPs and assessed the strength of our instrumental variables using the *F* statistic (beta^2^/se^2^). An F-statistic exceeding 10 was considered indicative of a robust instrument [21]. *R*^2^ was calculated as beta^2^/[beta^2^ + se^2^*(*N*− 2)], *N* being the sample size, and the genetic variability explained by each SNP was calculated [22]. Finally, after eliminating palindromic SNPs, we proceeded to utilize the remaining selected SNPs as our instrumental variables for subsequent analyses. To delve into the direct influence of distinct autoimmune diseases on sepsis, we adopted a multivariate MR approach-an extension of the conventional univariate MR. This approach duly acknowledged the inherent interplay among SNPs used in MR analyses, often manifesting shared associations across different autoimmune conditions. In our study, the SNPs utilized for multivariate MR were formulated as combinations of instrumental variables per exposure, thereby accounting for the intricate web of associations (including those associated with phenotypes of at least two autoimmune diseases). This study is reported in line with the STROBE-MR guidance, with the checklist available in the Supporting information [23].

#### Data sources for exposures, mediators and outcomes

Recognizing that utilizing diverse populations could potentially lead to biased estimates, we constrained the genetic background of the population in the MR study to individuals of European ancestry [24].

**Exposures** included a total of 10 distinct autoimmune diseases, and we aligned our analysis with data available from the FinnGen consortium (R9) (https://www.finngen.fi/fi*.*) and the MRC-IEU online database (https://gwas.mrcieu.ac.uk/). The autoimmune diseases considered for inclusion in our analysis were as follows: systemic lupus erythematosus (SLE), ankylosing spondylitis, multiple sclerosis (MS), primary biliary cholangitis, rheumatoid arthritis (RA), Crohn's disease (CD), ulcerative colitis (UC), type 1 diabetes (T1DM), celiac disease, psoriasis. We systematically reviewed and summarized the general characteristics of each autoimmune disease, and we presented these aggregated data in Additional file 3: Table S1. It is important to note that the ten autoimmune diseases from the Finngen consortium were defined using the codes of the International Classification of Diseases (ICD-9) and ICD-10. In the FinnGen consortium, individuals with undefined sex, high genotype deletion (> 5%), excess heterozygosity (± 4 standard deviations ((SDs)), and non-Finnish ancestry were excluded. All genetic association effect sizes were calculated by logistic regression, and adjusted for age, sex, and genetic principal components [25]. In the MRC-IEU database, all 10 included diseases have been previously published online. However, due to their diverse origins from different research teams, the analytical methods employed and the controlled confounding factors are not entirely uniform. For a comprehensive understanding, please refer to the cited references for detailed information [26–34].

**Mediators** On the basis of literature reviews of observational and MR studies, we selected 21 candidate mediators [35] (Additional file 3: Table S2), including blood cell counts, immunoglobulin levels, and serum cytokine concentrations that may be altered by autoimmune diseases, according to four criteria [36]. First, on the basis of collective scientific knowledge, candidate mediators might lie on the pathways from autoimmunity to sepsis. Second, candidate mediators were potentially accessible clinical interventions. Third, the GWAS data for candidate mediators should be available in individuals of European ancestry or predominantly European ancestry from large-scale consortia or cohorts with no or merely mild sample overlap with the GWASs of exposures and outcomes. Blood cell counts include: basophil cell count, eosinophil cell count, lymphocyte cell count, monocyte cell count, neutrophil cell count, white blood cell count; serum immunoglobulin levels included IgA, IgM, IgG, IgE; cytokines included IL-1α, IL-6, IL-12, IL-4, IL-10, IL-13, TGF-β1, TGF-β2, TGF-β3, TNF-α, and IFN-γ. We screened for mediators of the causal relationship between autoimmune disease and sepsis based on the following criteria: (1) the mediator should be causally related to sepsis; (2) the mediator should have a direct causal effect on sepsis independent of the autoimmune disease; (3) the autoimmune disease should be causally related to the mediator rather than the other way around; and (4) the association of the autoimmune disease with the mediator and the association of mediators with longevity outcomes should be in the opposite direction[37]. The genetic instruments for each mediator were at a genome-wide significance level (*P* < 1.00 × 10^–5^) independent of each other (LD *r*^2^ < 0.001 within 10,000 kb).

**Outcomes** Sepsis (ID: finn-b-AB1_SEPSIS) was built by the FennGen consortium and included 203,824 Europeans (6164 cases and 197,660 controls) with 16,380,410 SNPs. Sepsis (28-day death, ID: ieu-b-5086) was built by the UK Biobank consortium and contained 486,484 Europeans (1896 cases and 484,588 controls) with 12,243,487 SNPs. ICD-10 codes A40, and A41 were used to identify sepsis[38]. These data performed were analyzed using regenie v2.2.4, adjusted for age, sex, chip, and the first 10 PCAs.

#### Other factors

To satisfy the second condition of MR, we conducted an exhaustive search within the PhenoScanner database, aiming to identify established links between instrumental SNPs and potential confounding variables. Given our focus on autoimmune diseases as the target exposures, it became evident that the most influential genetic loci associated with these conditions were predominantly situated within the major histocompatibility complex (MHC), a genomic region intricately involved in adaptive immune responses. However, due to the robustness of the associations between variants in the MHC region and autoimmune diseases, as well as the intricate structure of linkage disequilibrium (LD) within this region, we took a deliberate step to exclude variants falling within the MHC region [39, 40]. Specifically, we defined this exclusionary region as encompassing base positions 24,000,000 to 35,000,000 on chromosome 6 (GRCh37). This precautionary measure was taken to mitigate the potential impact of strong associations with autoimmune diseases and the intricate LD structure, which could introduce horizontal pleiotropy and undermine the assumptions central to the MR framework. Subsequently, we conducted a fresh round of MR analyses, this time excluding instrumental variables linked to the MHC, with the intention of minimizing the influence of confounding factors.

#### Statistical analyses

We used the Wald ratio to estimate the effect of exposure on outcome for each IV and then adopted the random effects inverse variance-weighted (IVW) method to combine each IV’s effect size [41]. In addition, MR-Egger and weighted-median methods were used as supplements to IVW. The weighted median specifies that at least 50% of the weight in the analysis comes from variables that are valid instruments, whereas the weighted mode requires that the largest subset of tools identifying the same causal effect be effective tools [42]. The Cochrane’s Q value was used to assess the heterogeneity. The MR-Egger intercept and MR-Pleiotropy Residual Sum and Outlier (MR-PRESSO) methods were used to detect horizontal pleiotropy [43, 44]. If the outliers were detected, they would be removed and we would reassess the MR causal estimation. The MR-PRESSO-corrected results are reported in the main results as well, as they adopted the IVW method. To account for multiple testing in our analyses, a Bonferroni-corrected threshold of *P* < 0.0025 (*α* = 0.05/20, 20 = 10 autoimmune diseases × 2 different sources) was applied. Associations with *P* < 0.0025 were considered significant, and associations with *P* ≥ 0.0025 and < 0.05 were considered suggestive. To mitigate potential confounding factors, a multivariable Mendelian Randomization study was conducted on the exposure that was causally associated with the outcome. Otherwise, to adjust for SLE in our models, we performed a multivariable IVW MR analysis. In multivariable MR (MVMR) analysis, the IVW model was also the main method and the MR-Egger method was the complementary method [45]. A fixed-effect model was used to combine the MR results derived from FinnGen and MRC-IEU database. All statistical analyses were conducted using the Mendelian Randomization (0.4.2), TwoSampleMR (0.5.7), MR-PRESSO (1.0), MVMR (0.3) and meta (4.11.0) packages in R, version 4.2.2 (https://www.r-project.org/).

### Real-world observational analysis

#### Data source

This study used the publicly available Multiparameter Intelligent Monitoring in Intensive Care (MIMIC) IV database version 1.0 [MIMIC-IV, a freely accessible electronic health record dataset][46]. A total of 523,740 admissions were recorded in the MIMIC-IV database, of which 76,540 were admitted to the ICU, and was jointly developed by the Massachusetts Institute of Technology, Phillips Healthcare, and Beth Israel Deaconess Medical Center.

#### Patient population

The primary study population consists of adult ICU patients with sepsis. All patients were required to have at least 24 h of ICU data, and we selected the last ICU stay meeting these criteria for each patient. We identified 2537 patients in the database meeting the autoimmune disease inclusion criterion. Throughout this study, autoimmune disease and sepsis (Based on the Sepsis 1.0–3.0 version) refers to a set of related conditions, defined using ICD-10-CM diagnosis codes and free text analysis of the patient discharge summaries. In addition, it should be noted that due to database limitations we may have difficulty distinguishing whether a patient was admitted to the ICU for sepsis or developed sepsis after being admitted to the ICU for an autoimmune disease. For each patient in the study, we extracted several confounding factors from data stored in the MIMIC-IV database. They included age, race, sex, Sequential Organ Failure Assessment (SOFA) score, steroid and Disease-modifying antirheumatic drugs (DMARDs), and Charlson-score at ICU admission. SOFA includes information about the condition of a patient’s respiratory, renal, and cardiovascular systems, among others, and has been found to be a strong predictor of prognosis for ICU patients with sepsis[47]. The detailed information on steroids and DMARDs is as follows: azathioprine, chloroquine, cyclosporine, penicillamine, auranofin, hydroxychloroquine, leflunomide, methotrexate, minocycline, sulfasalazine, tofacitinib, and cyclophosphamide. The primary outcome of interest in this study is patients’ occurrence and 28-day mortality of sepsis. The 28-day mortality rate is based on data from the Social Security Death Index, which reflects the number of deaths within the 28-day window after discharge and the number of hospital deaths.

#### Statistical analysis and modeling

In the analysis, we estimated relative risks with odds ratios (ORs) and 95% confidence intervals (CIs) for patients with sepsis, compared with patients without sepsis using a multivariable logistic regression model [48]. ORs were adjusted for potential confounders using two approaches: (1) all potential confounders were included in the final model and (2) only potential confounders that meaningfully affected model estimates were included in the final model. Factors that were considered as potential confounders included the following: age, race, SOFA score, Charlson-score, steroids and immunosuppressants. In this analysis, we used multivariable logistic regression model with both confounder adjustment approaches (discussed above) and report the odds ratio (OR). We performed all statistical analyses using the SPSS software (IBM Corporation, version 26). *P* < 0.05 was regarded as significant.

## Result

### Mendelian randomization

The F statistics for IVs and estimated power for all analyses are shown in Additional file 3: Table S3-S4. None of these IVs had an F-statistic below the threshold of 10, and the explained variances varied from 0.16 to 71.8%. No pleiotropy was identified in the analysis of all exposures in the MRC-IEU and FinnGen consortium by the MR-Egger regression. Mild heterogeneity was found in the analysis of some exposures (*P* for Cochrane’s *Q* < 0.05) (Additional file 3: Table S5). Using MR-PRESSO to detect outliers, although in some computations Global test *P* < 0.05, but we did not identify any outliers.

**Fig. 2:**
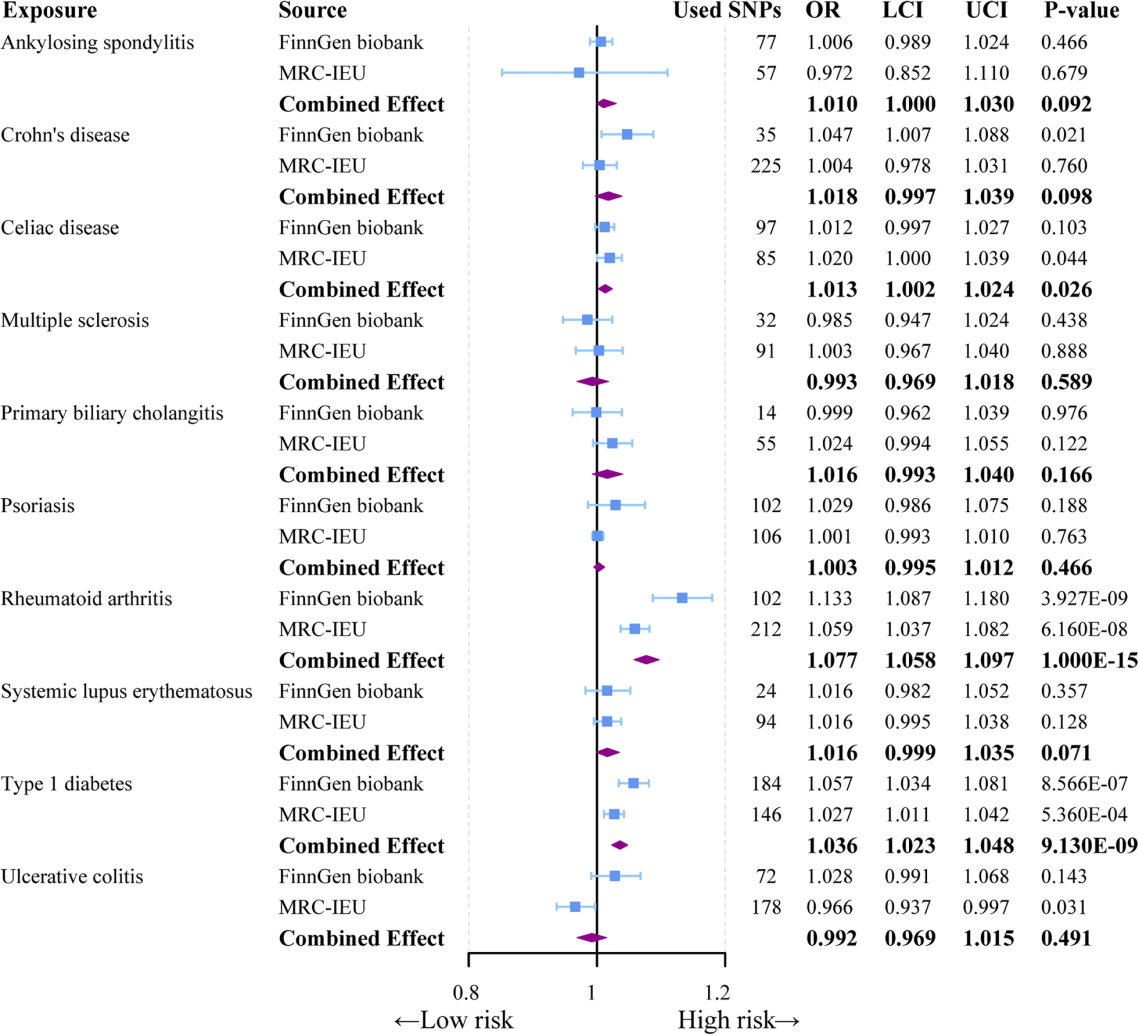
Forest plot to visualize the causal effect of autoimmune diseases (non-MHC loci SNPs) on sepsis using the inverse variance-weighted method and meta-analysis. CI: 95% confidence interval. OR, odds ratio

**Fig. 3:**
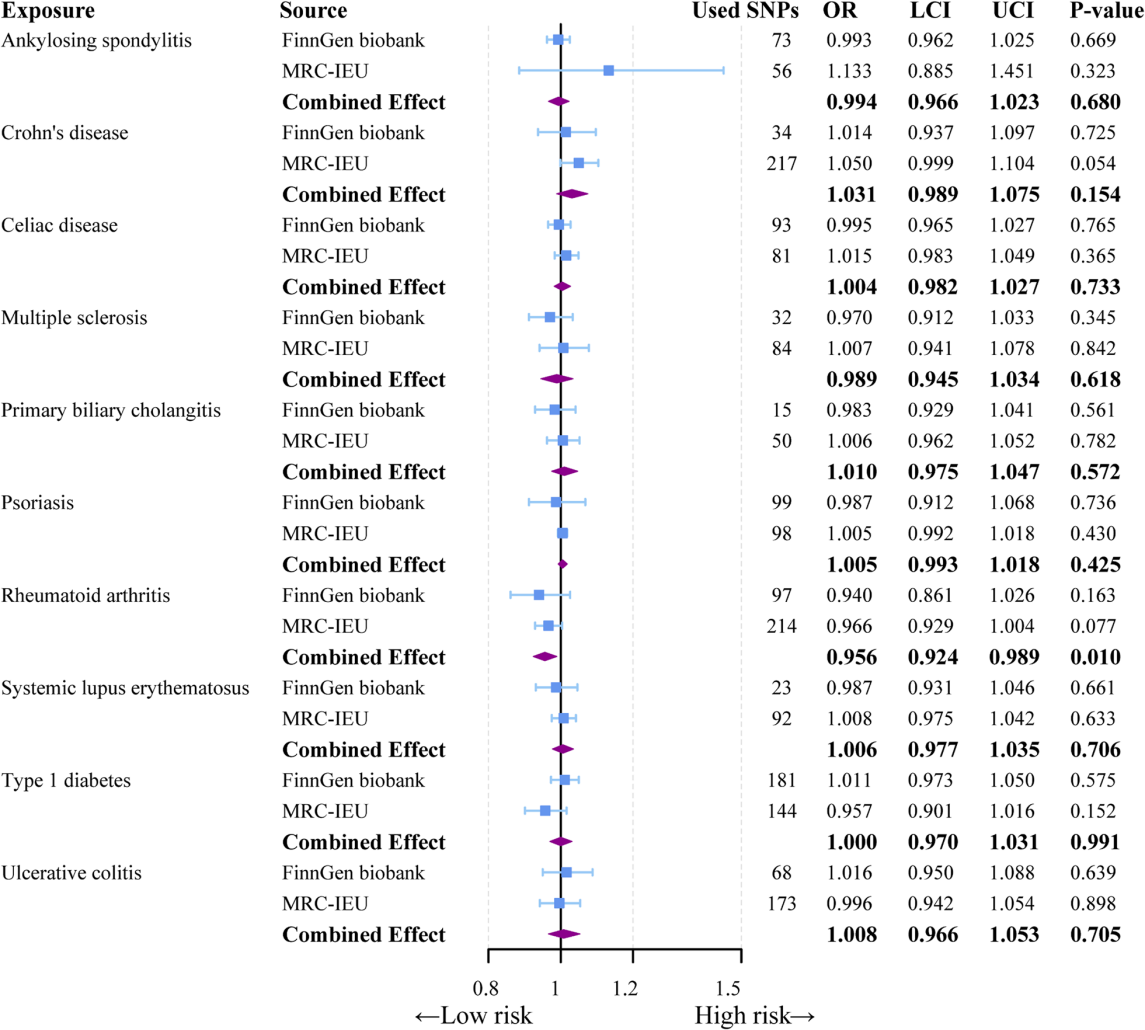
Forest plot to visualize the causal effect of autoimmune diseases (non-MHC loci SNPs) on sepsis 28-day mortality using the inverse variance-weighted method and meta-analysis. CI: 95% confidence interval. OR, odds ratio

In the univariable MR study, there was a causal association between genetically predicted RA (OR = 1.077, 95% CI = 1.058–1.097, *p* = 1.00E-15) and T1DM (OR = 1.036, 95% CI = 1.023–1.048, *p* = 9.130E-09) with sepsis. This connection holds true for exposure data from both the FinnGen consortium and the MRC-IEU database. Moreover, this association is nearly consistent across two additional complementary analytical approaches (MR-Egger and Weighted Median), as demonstrated in the Additional file 3: Table S5. The genetic-level association between celiac disease and sepsis varies between the FinnGen consortium and MRC-IEU databases. However, following the meta-analysis, the P_IVW_ combined effect size < 0.05, suggesting a potential genetic-level connection between celiac disease (OR = 1.013, 95% CI = 1.002–1.024, *p* = 0.026) and sepsis (Fig. 2). Conversely, for CD (OR = 1.018, 95% CI = 0.997–1.039, *p* = 0.098) and UC (OR = 0.992, 95% CI = 0.969–1.015, *p* = 0.491), genetic-level disparities with sepsis are evident in databases from two distinct sources. However, the post meta-analysis yielded *P*_IVW_ values > 0.05, indicating no significant association with sepsis (Fig. 2). Surprisingly, despite an increasing trend in sepsis for most diseases, we did not identify a significant genetic-level association between several other autoimmune diseases (SLE, ankylosing spondylitis, multiple sclerosis, primary biliary cholangitis) and sepsis (Fig. 2). Furthermore, we explored the causal relationship between autoimmune diseases and 28-day sepsis-related mortality. Unexpectedly, our study did not find any significant association between the included autoimmune diseases and 28-day sepsis-related mortality, except for the potential negative correlation between RA and sepsis-related mortality (Fig. 3, Additional file 3: Table S7). In addition, we further analyzed the association of IVs of autoimmune diseases containing MHC loci with sepsis and its 28-day mortality. Compared to the MR analysis without MHC loci, the same causal association between celiac disease and sepsis (OR = 1.019, 95% CI = 1.008–1.030, *p* = 5.000E-4) was observed in the MR analysis including SNPs at MHC loci, except for RA and T1DM (Additional file 2: Fig. S1, Additional file 3: Table S6). However, no causal association was found for autoimmune diseases in the MR analysis including MHC loci with sepsis 28-day mortality as an outcome (Additional file 2: Fig. S2, Additional file 3:Table S8).

**Fig. 4:**
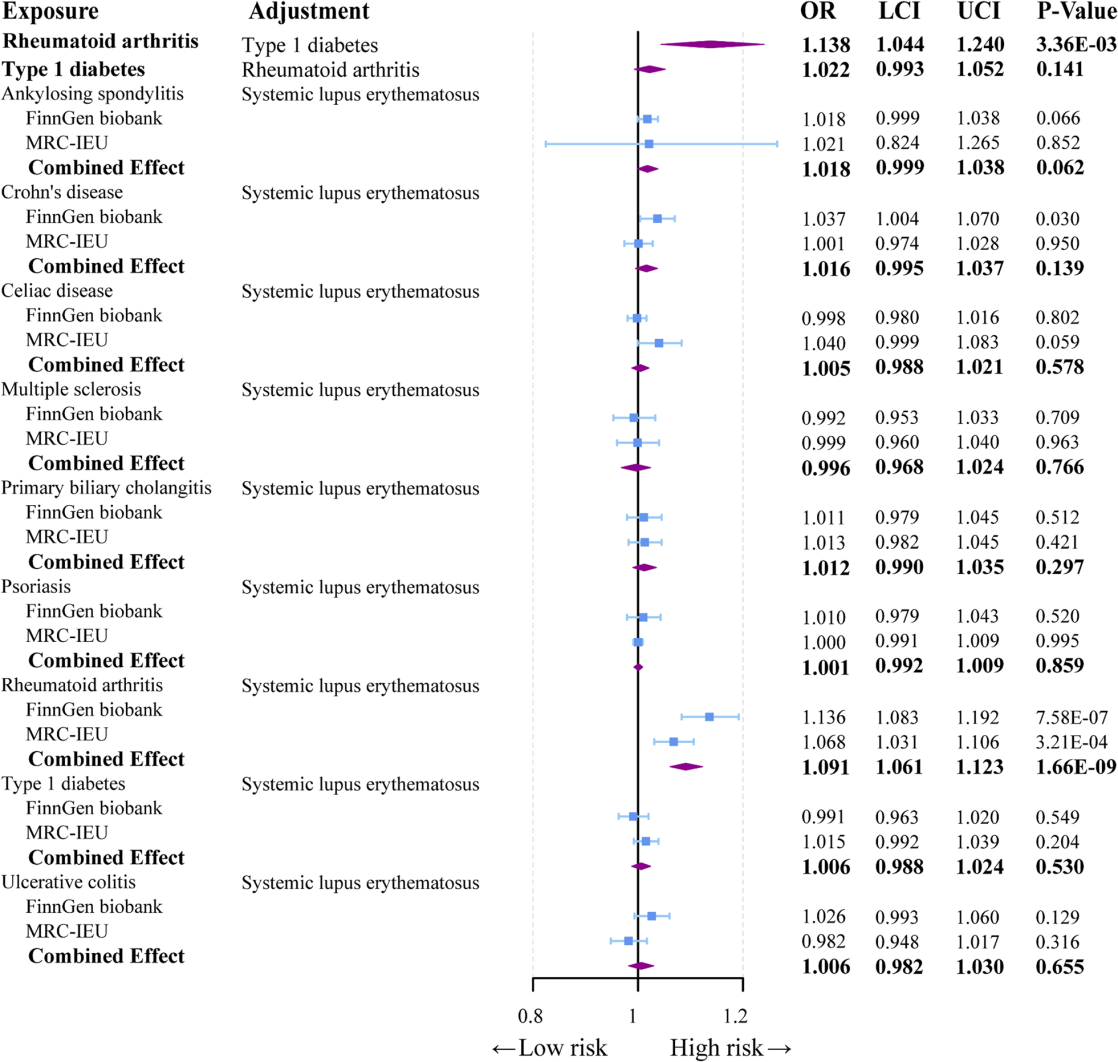
Forest plot to visualize the associations of genetically predicted autoimmune diseases with sepsis using multivariable MR analyses and meta-analysis. CI: 95% confidence interval. OR, odds ratio

In the multivariable MR analysis, we conducted analyses on autoimmune diseases potentially associated with sepsis in the FinnGen consortium dataset. Surprisingly, following the multivariable MR analysis, only RA (OR = 1.138, 95% CI = 1.044–1.24, *p* = 3.36E-03) was found to have a causal association with sepsis (Fig. 4, Additional file 3: Table S9). Using PhenoScanner, we identified associations of instrumental variables (IVs) with SLE and other autoimmune diseases. Consequently, we performed a multivariable analysis with other exposure factors and SLE to explore more stable exposure factors causally related to sepsis. After multivariable adjustment, we found that RA (OR = 1.091, 95% CI = 1.061–1.123, *p* = 1.66E-09) continues to exhibit a causal association with sepsis by adjusting for SLE (Fig. 4). In summary, based on the comprehensive analysis, we can deduce that RA is independently associated with the risk of sepsis occurrence.

**Fig. 5:**
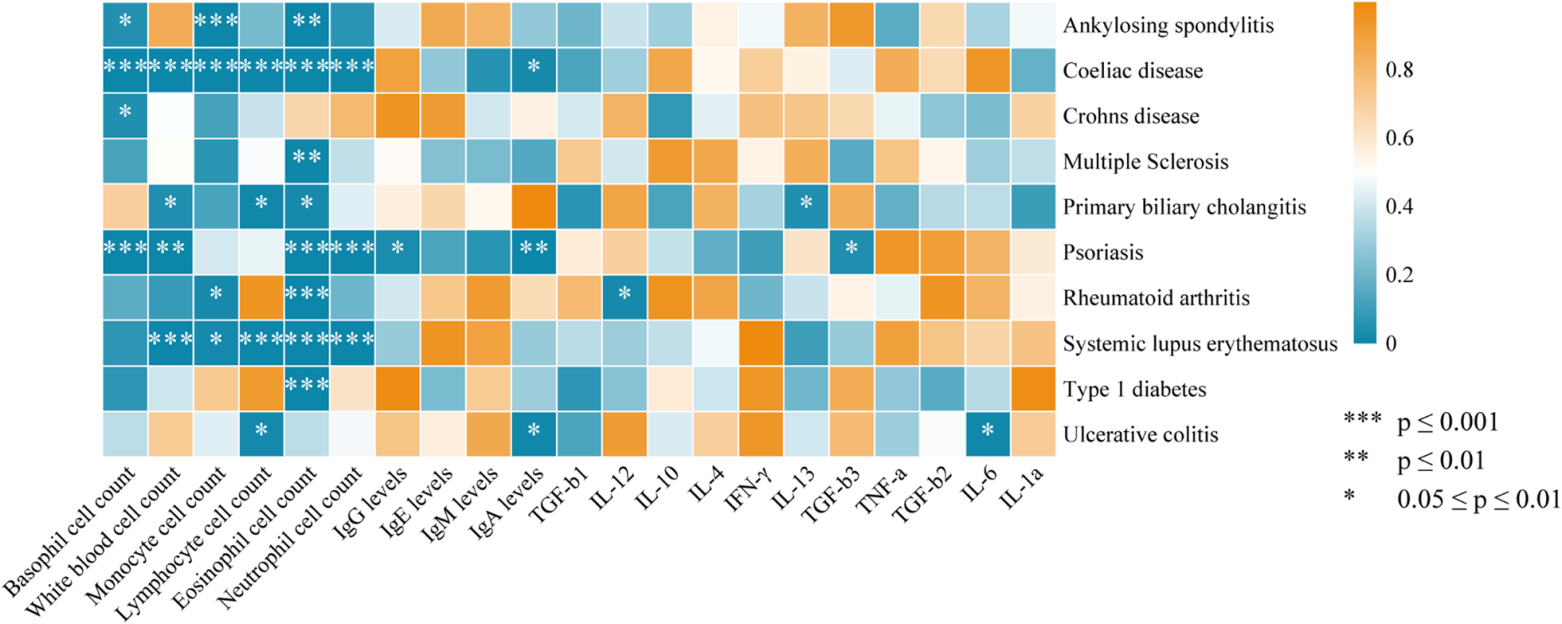
The heat map displays the MR analysis results of autoimmune diseases and mediators

**Fig. 6:**
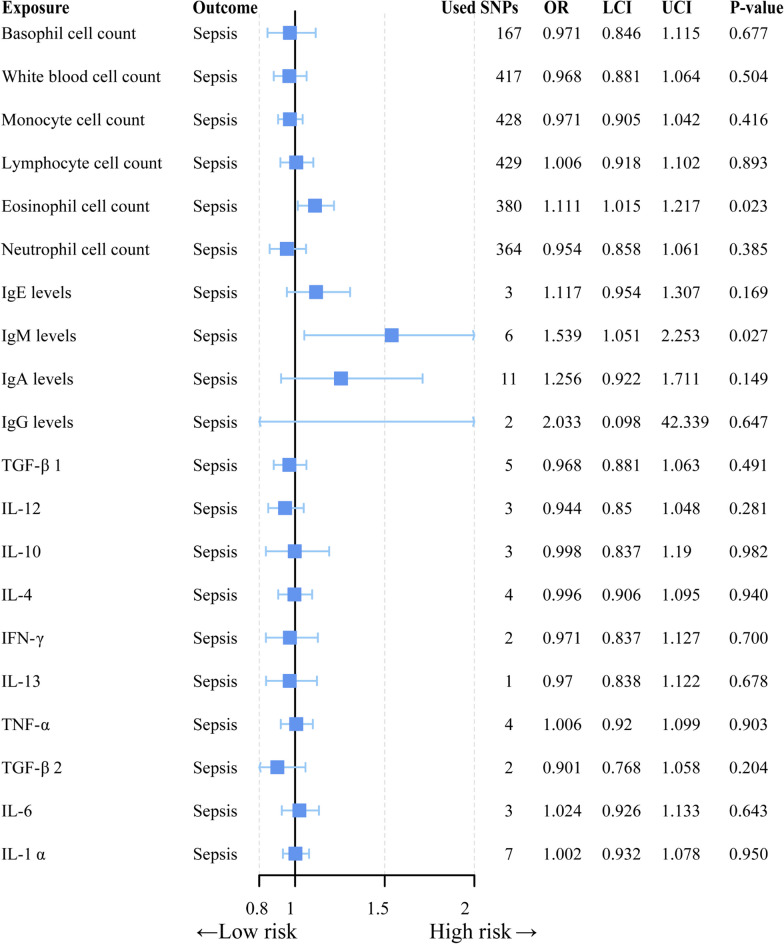
Forest plot to visualize the causal effect of mediators on sepsis using the inverse variance-weighted method. CI: 95% confidence interval. OR, odds ratio

To assess the potential induction of sepsis by autoimmune diseases through latent mediating factors, we incorporated possible sepsis-related risk factors, including blood cell counts, immunoglobulins, inflammatory cytokines, and more, based on literature [49]. We screened for eligible mediating factors according to established criteria. Regrettably, we did not discover any mediating factors between autoimmune diseases and sepsis (Fig. 5, Additional file 3: Table S10). Our mediation analysis results indicated that certain autoimmune diseases could lead to decreased blood cell counts (e.g., SLE, celiac disease). However, we did not observe a causal relationship between blood cell counts and sepsis occurrence (Fig. 6, Additional file 3: Table S11). Therefore, the reduction in blood cell counts induced by autoimmune diseases is not causally linked to sepsis development. Similarly, the levels of immunoglobulins and autoimmune disease-related cytokines also failed to mediate the relationship between the two (Figs. 5, 6, Additional file 3: Table S12–S15). Hence, the mediation analysis further reinforces the absence of a widespread causal relationship between autoimmune diseases, their potential correlates, and the occurrence of sepsis.

**Fig. 7:**
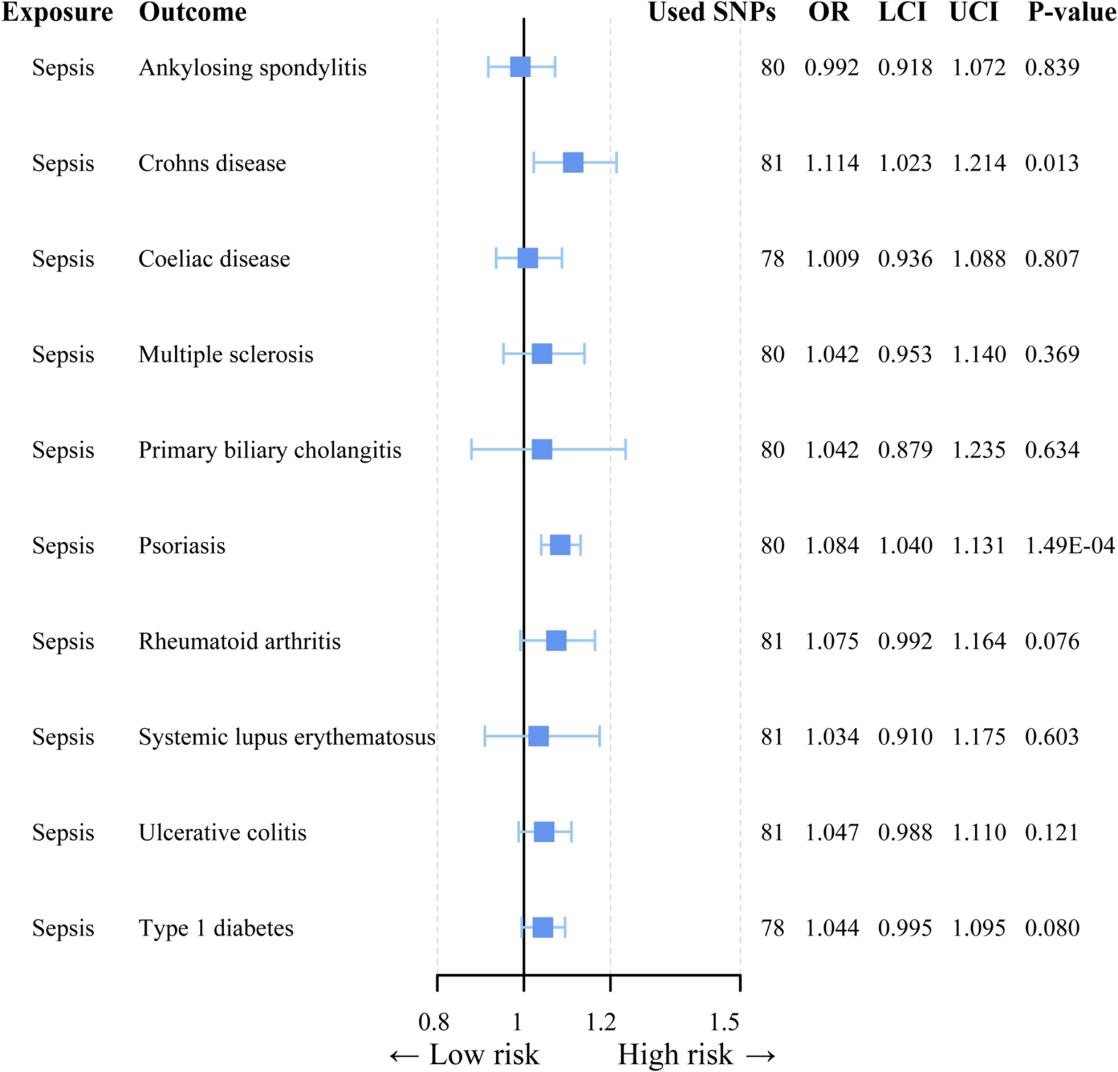
Forest plot to visualize the causal effect of sepsis and autoimmune diseases using the inverse variance-weighted method. CI: 95% confidence interval. OR, odds ratio

Sepsis is a severe infectious disease caused by pathogenic microorganisms, and immune dysfunction stands out as one of its primary characteristics. Previous studies have indicated a correlation between microbial infections and the onset of autoimmune diseases, as well as the significance of immune dysfunction as a triggering factor for autoimmune conditions [50, 51]. Hence, we explored the potential causal relationship between sepsis and autoimmune diseases at the genetic level. Through reverse MR, we discovered a causal association between genetically predicted sepsis and the occurrence of psoriasis (OR = 1.084, 95% CI = 1.040–1.131, *p* = 1.488E-04), while no associations were observed with other autoimmune diseases (*p* = 0.05/10) (Fig. 7, Additional file 3: Table S16, S17).

### Observational study

The study cohort consisted of 2537 patients with autoimmune disease. Among them, 1176 (46.0%) developed sepsis. The baseline characteristics for patients with sepsis and without sepsis are shown in Table 1. Generally, autoimmune diseases combined with sepsis tend to have higher SOFA scores, Charlson scores, steroid use, and a higher risk of death.

**Table 1:** Baseline characteristics of autoimmune disease patients

	Sepsis	Non-sepsis	*P* value
Number of patients	1167	1370	
Patient outcomes			
28-day mortality	18.1%	8.3%	0.002
Patient characteristics			
Age (mean ± SD)	66.42 ± 16.52	63.50 ± 17.93	< 0.001
Sex (% Female)	42.6%	46.0%	< 0.001
Race			< 0.001
Asian	2.6%	3.2%	
Black	8.2%	10.1%	
Hispanic	3.3%	3.6%	
White	66.9%	67.4%	
Other	4.0%	4.0%	
Unknow	14.5%	11.9%	
SOFA at admission (mean ± SD)	6.74 ± 3.78	3.07 ± 2.58	< 0.001
Charlson score	6.59 ± 3.02	5.85 ± 3.06	< 0.001
Chronic prednisone use	12.5%	8.8%	< 0.001
Chronic DMARDs use	0.7%	0.6%	0.149

A total of 1167 (46.0%) patients with sepsis and 1370 (54.0%) patients without sepsis in autoimmune disease patients. In our analysis, we employed a multivariate logistic regression model to adjust for various potential confounding factors, such as age, SOFA score, ethnicity, Charlson score, etc., to explore the relationship between autoimmune diseases and sepsis, and the 28-day mortality from sepsis in a more robust manner. After adjustment, rheumatoid arthritis (OR = 1.34, 95% CI = 1.11–1.64, *p* = 0.003) and multiple sclerosis (OR = 1.31, 95% CI = 1.03–1.68, *p* = 0.02) were associated with a higher risk of sepsis (Table 2). However, the presence of autoimmune diseases did not increase the risk of 28-day mortality in sepsis (Table 3).

**Table 2:** The relationship between autoimmune diseases and the occurrence of sepsis

Autoimmune diseases	OR (95% CI)	*P*-value
Systemic lupus erythematosus	1.16 (0.83–1.62)	0.38
Rheumatoid arthritis	1.34 (1.11–1.64)	0.003
Crohn’s disease	0.97 (0.65–1.44)	0.76
Psoriasis	1.26 (0.87–1.81)	0.06
Type 1 diabetes	1.04 (0.90–1.21)	0.57
Ankylosing spondylitis	0.74 (0.33–1.61)	0.40
Celiac disease	0.72 (0.38–1.32)	0.29
Multiple sclerosis	1.31 (1.03–1.68)	0.02
Ulcerative colitis	1.15 (0.85–1.56)	0.33

**Table 3:** The relationship between autoimmune diseases and 28-day mortality of sepsis

Autoimmune diseases	OR (95% CI)	*P*-value
Systemic lupus erythematosus	0.96 (0.50–1.87)	0.91
Rheumatoid arthritis	0.90 (0.62–1.30)	0.57
Crohn’s disease	0.69 (0.40–1.57)	0.38
Psoriasis	1.80 (0.96–3.34)	0.07
Type 1 diabetes	0.81 (0.59–1.12)	0.22
Ankylosing spondylitis	0.51 (0.15–1.40)	0.53
Celiac disease	1.08 (0.29–3.91)	0.91
Multiple sclerosis	0.70 (0.39–1.24)	0.22
Ulcerative colitis	1.03 (0.60–1.77)	0.90

## Discussion

In this study, we conducted a comprehensive investigation into the relationship between autoimmune diseases and sepsis, along with their 28-day mortality, employing both MR and real-world observational analyses. The results from the MR study indicate that only genetically predicted RA is causally associated with the occurrence of sepsis. Although there is a causal association between celiac disease and sepsis in MR analysis containing MHC loci SNPs, but we cannot rule out the possibility of pleiotropy leading to false positive results. Additionally, we found no mediators that could have mediated the potential association between autoimmune diseases and sepsis. Also, there was no causal association between autoimmune diseases and 28-day mortality from sepsis. In reverse MR analyses, a potential causal relationship between psoriasis and sepsis occurrence was detected. In the subsequent real-world study, after adjusting for factors such as age, SOFA score, Charlson score, and others, observational studies have found that only MS and RA increase the susceptibility to sepsis, and there was no association between selected autoimmune diseases and 28-day mortality in sepsis.

Over the past two decades, SLE has become one of the most prevalent autoimmune diseases in the ICU [15]. Among these cases, severe infections stand out as the most common cause of ICU admission and mortality [52]. While several retrospective studies have found a strong association between SLE and severe infections, this relationship is often multifactorial, and influenced by factors such as the use of moderate to high-dose corticosteroids, disease activity, and coexisting organ dysfunctions [53, 54]. Julia et al. demonstrated that the occurrence of infections, including severe infections, among SLE patients is correlated with immunosuppressive or immune-modulating medications [55]. In the context of other autoimmune diseases, such as RA, Bella et al.'s research revealed a significant increase in the incidence of sepsis when compared to non-inflammatory rheumatic and musculoskeletal diseases. This association persisted even after adjusting for other risk factors [56]. We also found that RA increases the occurrence of sepsis in patients. Likewise, studies by Ashwin et al. highlighted an elevated risk of sepsis among inflammatory bowel disease patients. However, this risk is intertwined with various factors such as age, comorbidities, and the use of immunosuppressive medications [57]. Similarly, Gary et al. identified independent factors associated with severe infections in CD, which included the use of prednisone, anesthesia pain relief medications, and moderate to severe disease activity [58]. In a retrospective study on MS, Richard et al. found a significant association between multiple sclerosis and severe infections [16]. In our study, MS also increased the susceptibility to sepsis. However, the occurrence of these infections was also strongly correlated with disease-related organ dysfunctions, such as decreased respiratory clearance capacity and bladder dysfunction [16]. Research by Iain et al. found that individuals with T1DM face a higher risk of severe infections compared to the general population. However, this outcome is influenced to varying extents by other contributing factors such as blood glucose levels, obesity, and age [59]. In the study conducted by Junko et al., revealed that the risk of severe infections among individuals with psoriasis is primarily linked to the severity of the disease itself. Moderate to severe psoriasis increases the risk of sepsis. However, this study did not specifically investigate the impact of biologic therapies [60]. In an observational study on ankylosing spondylitis, no significant correlation was found between ankylosing spondylitis and severe infections. This study concluded that ankylosing spondylitis does not have a significant relationship with severe infections, especially when considering the use of TNF blockers [61].

From the collective results of the mentioned studies and our study results, RA and MS increase the occurrence of sepsis. However, this association is often intertwined with factors such as the use of immunosuppressive medications, disease-related comorbidities, and age. Therefore, we conducted a two-sample MR analysis to investigate the relationship between autoimmune diseases and sepsis. Our study findings indicate that genetically predicted risk of SLE, CD, UC, MS, primary biliary cirrhosis, and psoriasis showed no causal relationship with sepsis. The occurrence of severe infections in these autoimmune diseases is likely more influenced by other contributing factors, such as the use of immunosuppressive/immunomodulatory medications, disease activity, comorbid organ dysfunctions, advanced age, and more. Therefore, we think that the increased occurrence of sepsis in MS may be closely related to the complications. While RA, T1DM showed causal associations with sepsis in univariable MR analysis, after conducting multivariable analysis, only RA remained causally associated with the occurrence of sepsis. This may be related to the existence of a common genetic basis for RA and sepsis. It has been found that abnormalities in PAD4 (Peptidyl arginine deiminase 4) structure and function lead to a significantly increased risk of developing RA, and that dysregulation of PAD4-mediated citrullination of extracellular proteins is a driver of the autoimmune response in RA, as 75% of patients develop anticitrullinated protein antibodies (ACPA) [62]. It is well known that Neutrophil extracellular traps (NETs) are one of the major factors contributing to the severity of septic disease, and that in the early stages of sepsis, depletion of NETs does not help to prevent or contain systemic infections, and even exacerbates pathological changes [63–66]. The citrullination of nuclear histones by PAD4 leads to chromatin depolymerization, which is a key step in the formation of NETs [67]. Therefore, the abnormal structure and function of PAD4 make RA patients more susceptible to sepsis. Chitinase-3 like-protein-1 (CHI3L1, other name YKL-40) may be another important protein molecule involved in the pathological process of RA and sepsis.YKL-40 is synthesized and secreted by a wide variety of cells including macrophages, neutrophils, and chondrocytes and plays an important role in tissue injury, inflammation, tissue repair and remodeling responses [68]. It was found that YKL-40 levels were significantly increased in RA patients and induced the expression of IL-1β and TNF-α, which were involved in the inflammatory response in RA [69]. A study by Kornblit et al. found that YKL-40 levels were also significantly elevated in patients with sepsis and that YKL-40 promoted the expression of inflammatory factors [70]. Thus, RA patients may be more susceptible to sepsis due to genetic variants, and the CHI3L1 genotype (rs4950928) may be a potential locus [69, 70].

In order to further explore the potential mediation of autoimmune diseases in the occurrence of sepsis through underlying intermediary factors, we included risk factors related to autoimmune diseases associated with sepsis [36]. We found that not all autoimmune diseases lead to a decrease in blood cell counts; only SLE, celiac disease, T1DM, and reduced blood cell count showed a causal association, whereas conditions such as RA and primary biliary cholangitis had less pronounced effects. Despite an inverse causal trend between blood cell counts and sepsis, statistical significance was lacking. While some observational studies suggest a predictive relationship between changes in blood cell counts and the risk of severe infection in autoimmune diseases [71, 72], these studies are often limited in their sensitivity and specificity. The cytokine storm is a prominent feature of sepsis, and autoimmune diseases frequently lead to alterations in cellular cytokine levels [73]. However, it remains unclear whether autoimmune diseases impact sepsis progression through cytokine modulation [74]. Through MR studies, we did not identify any potential associations, indicating that autoimmune diseases do not causally influence the occurrence of sepsis via changes in inflammatory cytokines. Similarly, we did not find a mediating role of immunoglobulin levels between the two conditions. Based on comprehensive mediation analyses, we concluded that factors such as blood cell counts, plasma inflammatory cytokines, and immunoglobulin levels do not mediate the causal relationship between autoimmune diseases and sepsis.

Considering the results from both univariate and mediation MR analyses, the relationship between autoimmune diseases and sepsis is likely the result of multiple overlapping factors. Therefore, the focus should extend beyond autoimmune diseases themselves, encompassing aspects like the use of immunomodulatory/suppressive drugs and comprehensive evaluation of organ function. Numerous studies on the use of biologics (such as Rituximab) in autoimmune diseases have shown an increased risk of severe infections [75–78]. Hydroxychloroquine reduces the risk of severe infections in SLE patients, while the use of glucocorticoids, especially in high doses, is closely related to severe infections [79]. Montgomery et al. found functional impairment to be a significant risk factor for severe infections in multiple sclerosis patients [17]. Therefore, patients with autoimmune diseases require closer monitoring of organ function, comorbidities, medication usage, and other factors to reduce the risk of sepsis. It is worth noting that RA is associated with an increased risk of sepsis, which can occur early in the course of the disease. This suggests that when managing patients with RA, early attention, timely treatment, and early prediction may be required to reduce the occurrence of severe infections. For example, for outpatient patients with fever or other signs of infection, infection-related markers and imaging tests should be monitored, and they should be hospitalized if necessary; for hospitalized patients, early monitoring of biomarkers and symptoms of infection, more active adjustment of antibiotics after infection, and more close monitoring for patients with high-risk factors (such as, indwelling catheters and steroid use, etc.) should be noticed.

The 28-day mortality risk in sepsis is a crucial measure of disease severity, and whether autoimmune diseases increase this risk remains inconclusive. A retrospective study by Antón et al. found that autoimmune diseases often lead to a higher mortality rate in critically ill patients [4]. However, this study primarily predicted high mortality risk without correcting for concurrent confounding factors such as SOFA score, age, underlying diseases, and had a relatively small sample size. In our analysis using two-sample MR analysis, we inferred causal relationships between autoimmune diseases and sepsis at the genetic level. We did not find a causal association between autoimmune diseases and the 28-day mortality rate in sepsis. Similarly, in the retrospective analysis from MIMIC-IV, there was no observed relationship between autoimmune diseases and the 28-day mortality rate in sepsis. Therefore, we believe that autoimmune diseases do not increase the 28-day mortality rate in sepsis. This might be related to the immune dysregulation caused by autoimmune diseases, leading to imbalanced cytokines in the sepsis inflammatory cascade [80], making it difficult to form a cascading reaction. The early mortality in sepsis is closely associated with this inflammation storm. Jorge et al.'s observational study found that the risk of death in autoimmune diseases may be related to factors such as experiencing shock upon admission to the intensive care unit, having hemoglobin levels below 8 g/dL, using immunosuppressive agents before ICU admission, and having low complement C3 levels [81]. Additionally, the quality of care provided by hospitals is a key factor influencing patient mortality risk, with more experienced hospitals often having lower mortality rates [3]. Therefore, for autoimmune disease patients admitted to the ICU, it is crucial to focus on the management of complications while enhancing diagnostic and treatment capabilities specific to autoimmune diseases to reduce the risk of mortality.

A key feature of sepsis is the immune dysfunction triggered by infections, leading to prolonged alterations in immune function such as changes in immune cell functionality and numbers. Similarly, the immunopathological mechanisms of autoimmune diseases are accompanied by disruptions in immune function [39, 82]. Furthermore, infections caused by pathogenic microorganisms can act as triggering factors for autoimmune diseases [83]. However, it remains uncertain whether the immune dysfunction triggered by severe infections caused by pathogenic microorganisms could lead to the development of autoimmune diseases. Through reverse MR analysis, we identified a causal relationship between sepsis and psoriasis, but no associations with other autoimmune diseases, and current research has also found that infection is an important trigger for the occurrence of psoriasis [84]. The specific mechanisms underlying this relationship require further investigation. MR provides a novel method to discover associations between different diseases at the genetic level, offering a new perspective for future observational studies.

This study has several limitations in the MR analysis. First, potential horizontal pleiotropy is a concern in any MR study. In our research, we did not observe significant evidence of pleiotropic effects in all exposure analyses using the MR-Egger intercept test. Additionally, the MR-PRESSO analysis detected few outliers, and associations remained consistent after removing outlier SNPs. However, the possibility of undetected outliers still exists. Second, sample overlap might be a concern as we selected a subset of autoimmune diseases from the FinnGen consortium. Nonetheless, sample overlap is unlikely to bias our results significantly, given that our IVs were selected from large-scale GWAS. Third, due to the limited number of SNPs meeting the inclusion criteria for certain autoimmune diseases (P value < 5e− 08, *R*^2^ = 0.001 with kb = 10,000), we slightly relaxed the selection criteria, which may introduce a certain level of false positives. Fourth, genetic factors are not the sole determinants of autoimmune disease onset; environmental factors also play a role in triggering disease processes. Therefore, our MR analysis lacks associations between genetically predicted autoimmune diseases and sepsis risk, but this does not exclude the potential impact of autoimmune diseases on the pathophysiology of sepsis [39]. Fifth, the genetic associations of blood cell count, inflammatory cytokines, and are based on relatively small global genomic studies, potentially leading to issues of statistical power. Sixth, univariate MR analysis may not capture the direct impact of specific biomarkers on disease outcomes, as the effect of a biomarker might be mediated by other biomarkers within a complex network. Seventh, all our analyses are based on individuals of European ancestry; generalizability to other populations requires further investigation. In real-world retrospective studies, there are also limitations. First, our results could be influenced by diagnostic bias, where the severity of autoimmune diseases and the immunocompromised state of autoimmune disease patients might lead them to be admitted to ICUs earlier than other populations, potentially resulting in better survival rates. This selection process could lead to a higher incidence of autoimmune diseases in the ICU population. Additionally, we controlled for potential influencing factors, yet the overall confounding factors included in our analysis might not be exhaustive, such as other clinical scores that were not incorporated. We believe that further research with more diverse pre-ICU admission data from intensive care units would help fully eliminate diagnostic bias. Second, the MIMIC database is derived from a single-center research institution, which may limit the generalizability of the study outcomes. Third, due to the limitations of the database, we were unable to distinguish whether patients with autoimmune diseases were diagnosed after their first ICU admission, i.e., it was difficult to determine whether the autoimmune disease was a newly diagnosed disease or a comorbidity, although this group of patients may be rare. Fourth, because Mendelian randomization uses integrated data, whereas observational studies use individual data, it is difficult for us to achieve correction for the same confounders for the two different analytical methods.

## Conclusion

In this study, our aim was mainly to assess the association of autoimmune diseases with the development of sepsis and 28-day mortality through a MR and observational study. In our results, genetically predicted RA was independently associated with the development of sepsis. We did not find that none of the other autoimmune diseases predicted by genes were independently associated with the development of sepsis, including subsequent mediation analyses. In addition, neither observational studies nor MR analyses found autoimmune diseases to be associated with 28-day mortality from sepsis., Surprisingly, there was a causal relationship between genetically predicted sepsis and the development of psoriasis.

### Supplementary Information

**Additional file 1:** STROBE-MR checklist.

**Additional file 2:** Figure.

**Additional file 3:** Table.

## Data Availability

The datasets analyzed in this study are publicly available summary statistics. Data used can be obtained upon a reasonable request to the corresponding author.
