# Supplementary material for: Association of autoimmune diseases with the occurrence and 28-day mortality of sepsis: an observational and Mendelian randomization study
Source: Crit Care. 2023 Dec 5;27:476. doi: 10.1186/s13054-023-04763-5 (PMC10698937; doi:10.1186/s13054-023-04763-5)
Supplement: Supplementary file 2 — Additional file 2: Figure. [file 13054_2023_4763_MOESM2_ESM.docx]

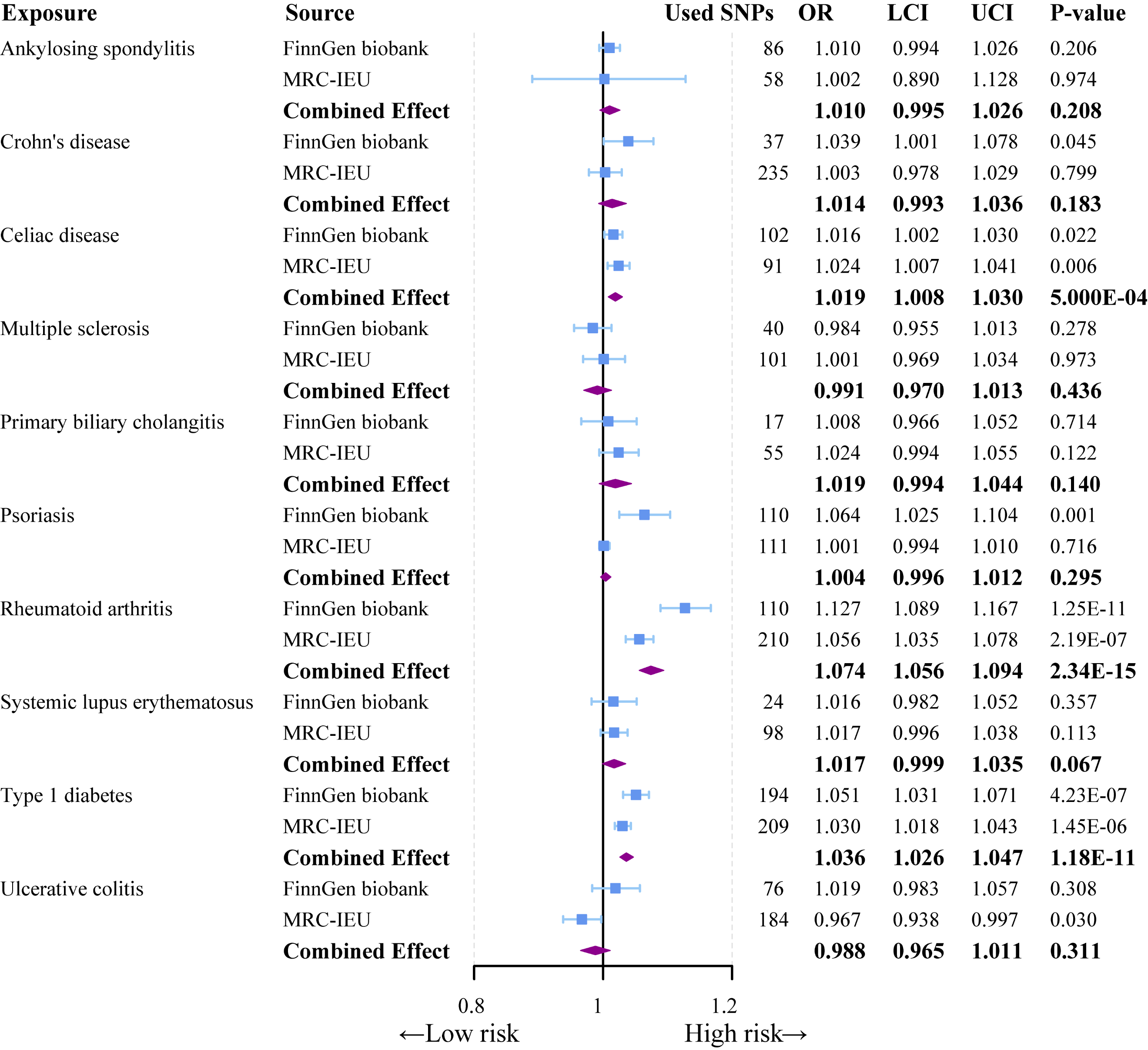


**Fig. S1** Forest plot to visualize the causal effect of autoimmune diseases (include-MHC loci SNPs) on sepsis using the inverse variance-weighted method and meta-analysis. CI: 95% confidence interval. OR, odds ratio.


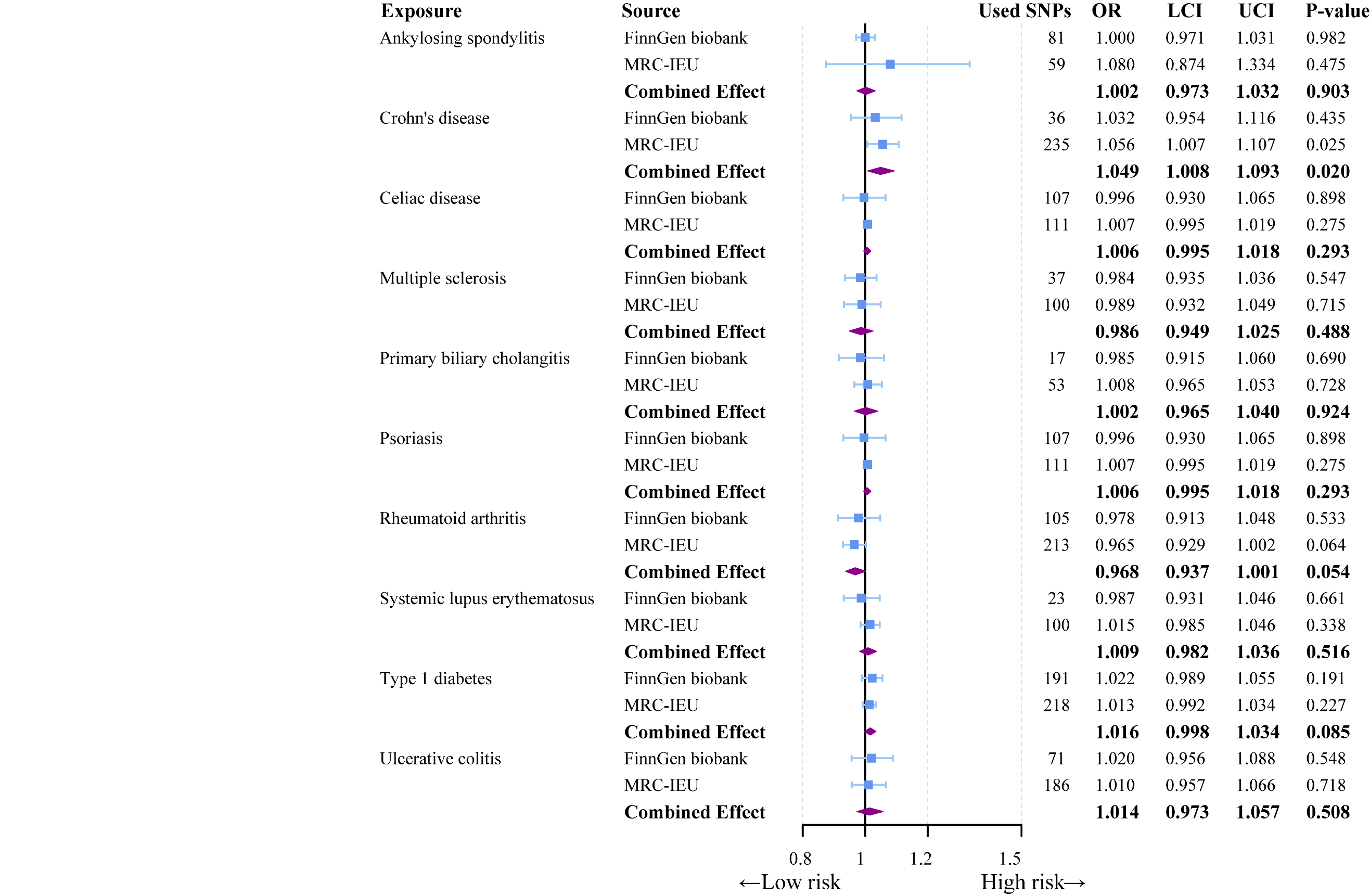


**Fig. S2** Forest plot to visualize the causal effect of autoimmune diseases (include-MHC loci SNPs) on sepsis 28-day mortality using the inverse variance-weighted method and meta-analysis. CI: 95% confidence interval. OR, odds ratio.
